# Superior peroneal retinaculum reattachment for an atraumatic peroneus brevis tendon subluxation: a case report

**DOI:** 10.1186/s13256-022-03455-y

**Published:** 2022-06-16

**Authors:** Camila Grandberg, Diovano Paust de Oliveira, Julio Cesar Gali

**Affiliations:** 1 Faculty of Medical and Health Sciences, Pontifical Catholic University of São Paulo, Rua Joubert Wey, 290, Sorocaba, SP Brazil; 2 Hospital Santa Lucinda, Rua Cláudio Manoel da Costa, 57, Sorocaba, SP Brazil; 3 Department of Surgery, Faculty of Medical and Health Sciences, Pontifical Catholic University of São Paulo, Rua Joubert Wey, 290, Sorocaba, SP Brazil

**Keywords:** Ankle joint, Atraumatic tendon subluxation, Case report, Peroneus brevis tendon, Superior peroneal retinaculum reattachment

## Abstract

**Background:**

Peroneal tendon subluxation is a rare pathology, generally associated with sport-induced trauma, that occurs due to the rupture of the superior peroneal retinaculum. The diagnosis is mainly clinical, but the use of imaging techniques, such as dynamic ultrasound and magnetic resonance imaging, may contribute to its clarification. Treatment may be conservative or surgical, although there is no consensus on the most appropriate technique to be employed. We report a case of subluxation of the peroneus brevis tendon, with no apparent traumatic cause, in which there was a need for a surgical approach after the failure of conservative treatment.

**Case presentation:**

A 25-year-old White woman presented pain and locking of the lateral side of the left foot 2 years earlier, with no history of trauma. The patient felt pain upon palpation and presented snapping during flexion–extension of the left ankle. On dynamic ultrasonography, an anterior subluxation of the peroneus brevis tendon occurred when the ankle was in dorsiflexion, suggesting superior peroneal retinaculum injury. Surgical correction was recommended after 2 months of conservative treatment with no improvement. The chosen surgical technique was isolated reattachment of the superior peroneal retinaculum, which proved successful.

**Conclusions:**

Peroneal tendon subluxation has no established preferred surgical technique. This case demonstrates superior peroneal retinaculum repair as an efficient surgical approach for this condition. Furthermore, the atraumatic mechanism of injury in this case, along with the unknown true incidence of peroneal tendon subluxation, highlights the need to consider this pathology in cases of ankle injuries.

## Background

Peroneal tendon subluxation is a relatively rare condition, and while it has become more frequent due to an increase in sports practice, it remains scarcely described in literature [1]. The mechanism of injury is inversion and dorsiflexion of the foot and ankle, simultaneously with an intense and abrupt contraction of the peroneal tendons [2].

Clinically, the subluxation presents with lateral ankle edema, pain, increased sensibility, and mobilization difficulty. The lesion may appear on initial examination or exacerbated through maneuvers, such as active and passive plantarflexion and eversion of the foot against resistance [3]. These maneuvers may demonstrate snapping, which consists of an audible click and projection of the tendons causing an abnormal dislocation of these structures over the distal fibula [4]. In recurrent subluxation cases, there may be a history of previous ankle lesions, possibly misdiagnosed as sprains, and sensation of instability and dislocation [5].

Although diagnosis is primarily clinical, some supplementary imaging methods, as high-resolution magnetic resonance imaging and ultrasound, are valuable. Dynamic ultrasound may show tendon subluxation during the dorsiflexion–eversion maneuver, so this is likely the most helpful tool for diagnosing peroneal subluxation, especially in cases of intermittent dislocation [4, 6].

Conservative treatment may be implemented in acute lesions, consisting of repositioning the tendons inside the retromalleolar groove, followed by immobilization of the distal portion of the leg [3]. However, acute subluxations may result in recurrent dislocations and become chronic subluxations, which have conservative treatment failure rates of over 50%. In these cases, as well as when tendons irreducibly dislocate in acute lesions, surgical treatment is recommended [1, 3].

## Case presentation

A 25-year-old White woman attended orthopedic consultation with pain and locking of the lateral side of the left foot 2 years before, with no history of trauma and no previous diagnosis. On examination, she felt pain upon palpation of the fifth metatarsal diaphysis and presented snapping during flexion–extension of the left ankle.

**Fig. 1:**
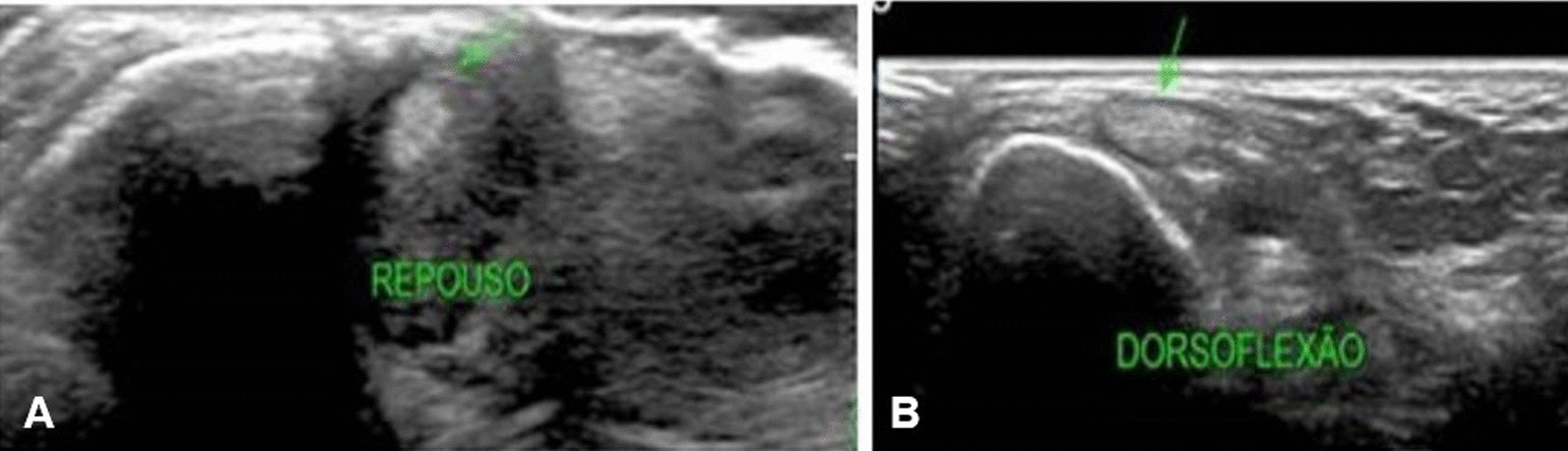
**A** Ultrasound of the left ankle, displaying reduced peroneus brevis tendon, in resting position (green arrow). **B** Subluxated peroneus brevis tendon during dorsiflexion (green arrow)

Ankle radiographs were considered normal, and dynamic ultrasonography demonstrated anterior subluxation of the peroneus brevis tendon with dorsiflexion, suggesting superior peroneal retinaculum injury (Fig. 1A, B).

After 2 months of conservative treatment, the condition did not improve. Therefore, surgical intervention was suggested. Having explained the procedure and its possible complications, the patient decided to proceed with the operative treatment.

**Fig. 2:**
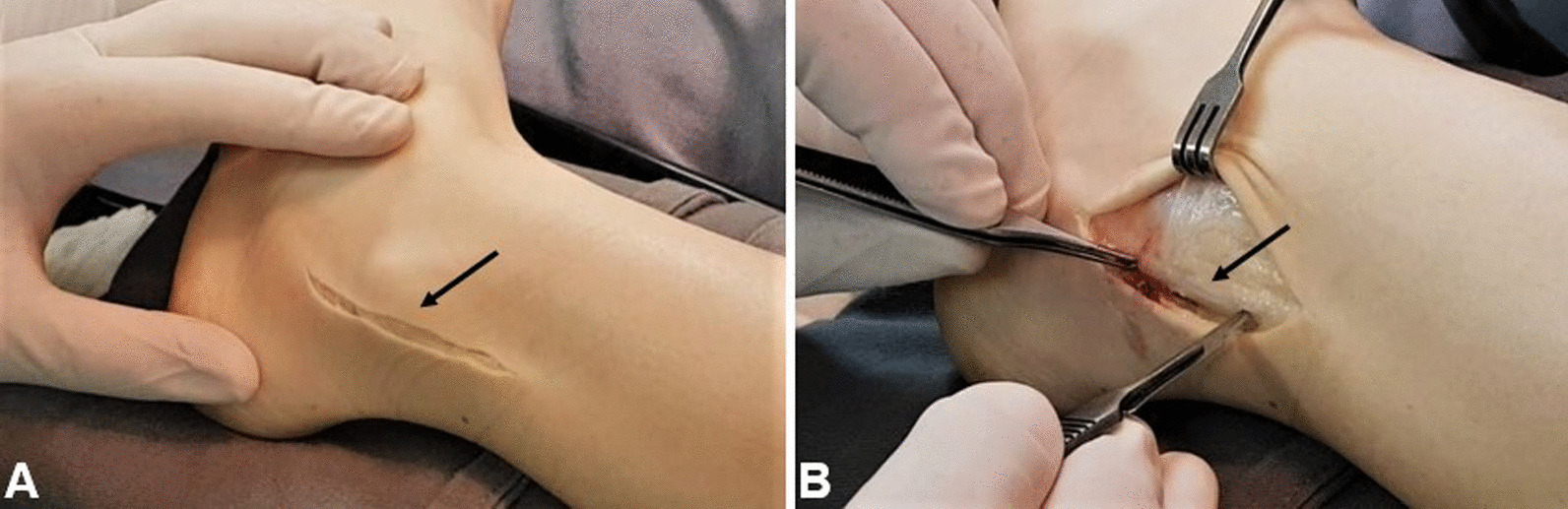
**A** Lateral aspect of the left ankle, showing surgical incision on the skin and subcutaneous (black arrow). **B** Opening of the peroneal tendons sheath (black arrow)

In this case, isolated anatomical reattachment of the retinaculum was performed, as described by Ferran *et al.* [5]. The patient was placed on the operating table in horizontal dorsal decubitus position, under spinal anesthesia, and a pneumatic tourniquet on the upper thigh, with a cushion under the buttock of the operative side. An approximately 6-cm longitudinal incision was made along the route of the peroneal tendons, and the peroneus brevis sheath was opened longitudinally around 3 mm posterior to the posterior border of the fibula (Fig. 2A, B).

**Fig. 3:**
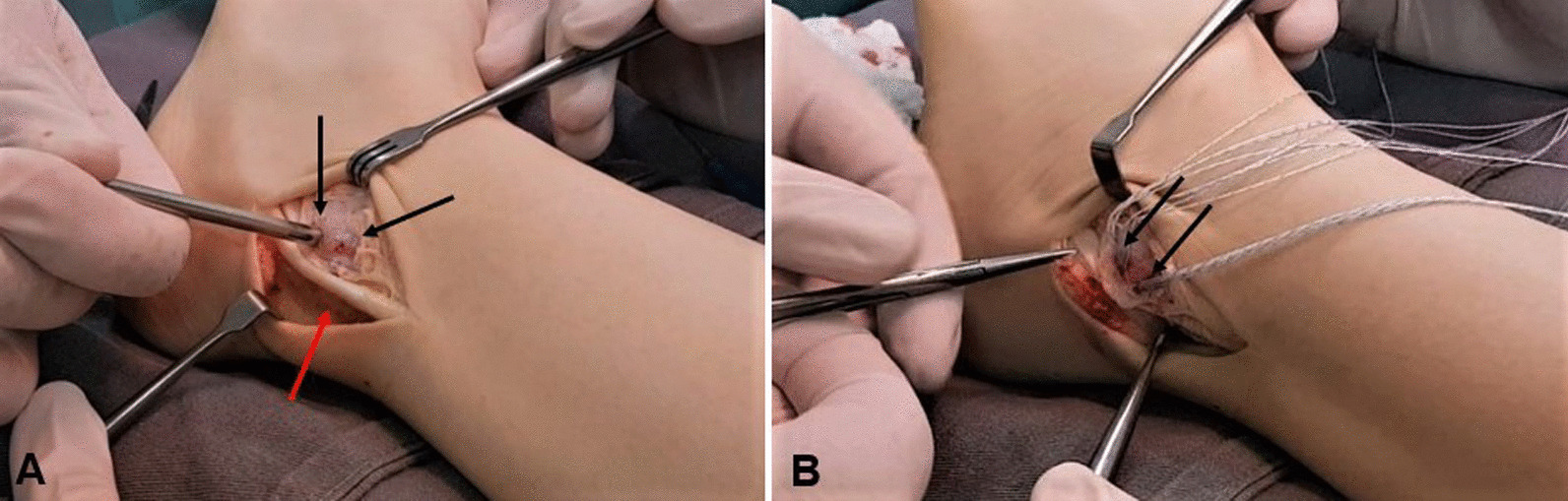
**A** Lateral aspect of the left ankle, demonstrating peroneus brevis tendon (red arrow) and creation of holes to place anchors (black arrows). **B** Inserted anchors and their sutures (black arrows)

**Fig. 4:**
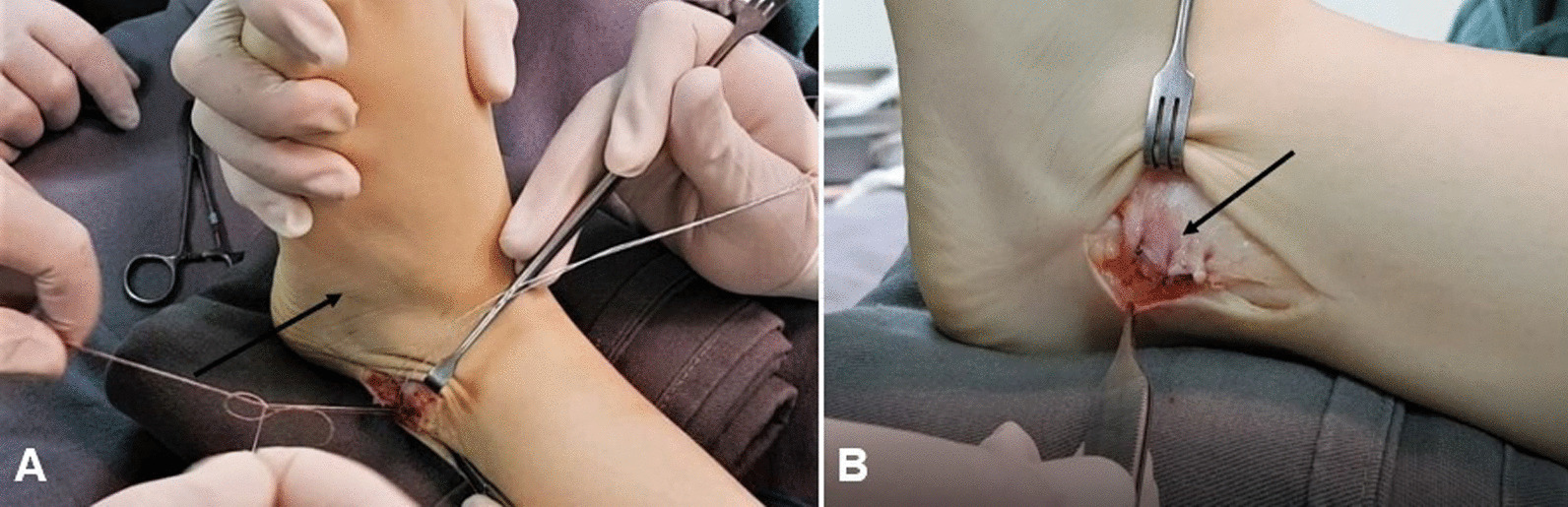
**A** Lateral aspect of the left ankle, exhibiting the joint kept in eversion and slight dorsiflexion while suturing to reattach the retinaculum (black arrow). **B** Lateral aspect of the left ankle showing “vest over pants” suture (black arrow)

Subsequently, the bony surface of the lateral malleolus was scraped until a bleeding surface was visible, and two anchors were inserted along the posterior border of the lower fibula (Fig. 3A, B). The posterior margin of the peroneal sheath was moved anteriorly, and the superior peroneal retinaculum was reconstructed by overlapping (“vest over pants” fashion), with the ankle in eversion and slight dorsiflexion. The strength of the repair was verified by moving the joint through its entire range of motion (Fig. 4A, B).

The surgical wound was closed, a crepe bandage was applied, and plaster immobilization was placed with the ankle in slight eversion and a neutral position of flexion and extension. The patient was discharged on the day after the surgery. After 10 days, the stitches were removed, and the ankle was immobilized with a synthetic cast for 4 weeks. Following its removal, partial weight-bearing was allowed, and physiotherapy was initiated. Cycling and swimming were introduced 2 weeks after the cast removal, and gradual stretching and strengthening exercises started after 8 weeks. Return to sports activities was authorized on the fifth postoperative month.

**Fig. 5:**
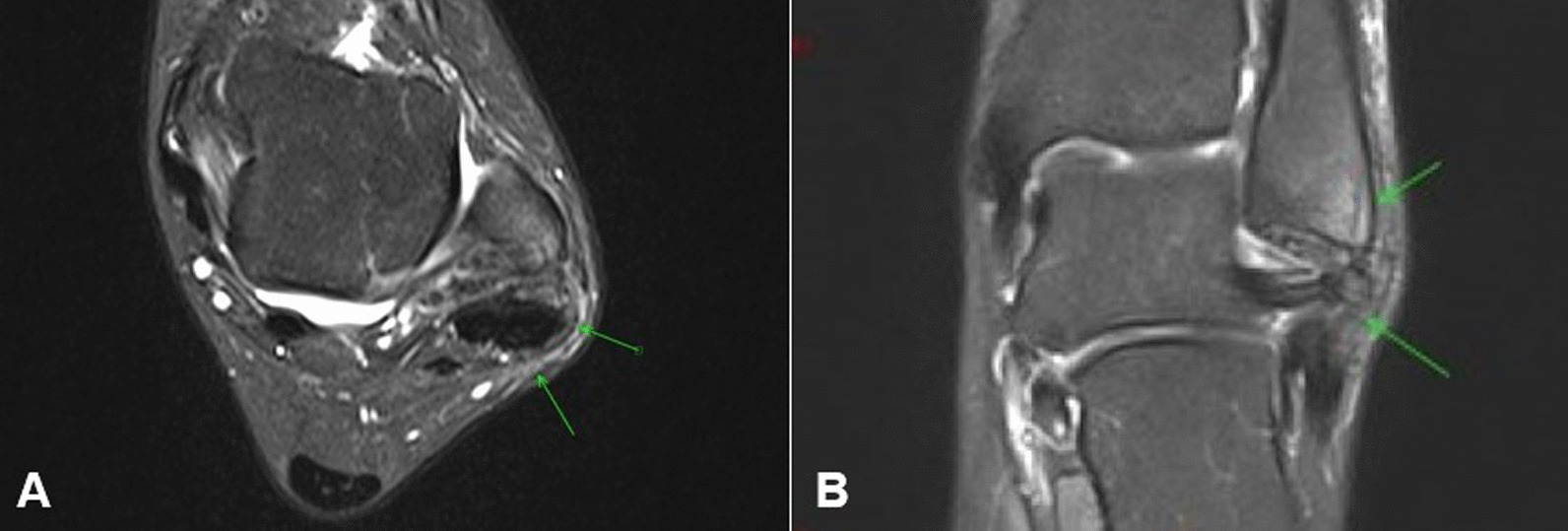
**A** Axial T2-weighted magnetic resonance imaging of left ankle, displaying repaired retinaculum (green arrows). **B** Coronal T2-weighted image showing the repaired retinaculum (green arrows)

There were no postoperative complications. Six months after surgery, magnetic resonance imaging was performed, where the reconstructed retinaculum was evident on T1 and T2 images (Fig. 5A, B). After the first postoperative year, the patient presented total ankle mobility, no subluxation recurrence, and no symptoms.

## Discussion and conclusions

The patient presented with sudden pain and locking of the left ankle without reporting previous trauma, although this injury typically occurs in traumas such as ankle sprains, and mainly in athletes who practice activities requiring shortcutting movements, such as skiing, ice-skating, soccer, basketball, rugby, and gymnastics [3, 7, 8]. However, retrospective studies suggest that this pathology may be asymptomatic in up to 35% of cases, in addition to being misdiagnosed as lateral ankle ligament sprains in up to 40% of cases, so its true incidence in clinical practice is unknown [9, 10].

Although there is no consensus on the most effective approach for this condition, positive outcomes have been reported with surgical interventions after failure of conservative treatment. The four main surgical techniques are reattachment or repair of the superior peroneal retinaculum, deepening of the retromalleolar groove, bone-block procedures, and tissue-transfer procedures [3].

Retromalleolar groove depth and shape have been described as possible predisposing factors for peroneal tendinopathies. Consequently, groove-deepening procedures may be performed, mainly when the sulcus is flat or convex [2, 11]. High complication rates have been associated with bone-block procedures, a technique that employs bone grafts as a physical barrier to the peroneal tendons, including redislocation, nonunion, tendon adherence to the bone, and chronic pain [11–13]. Tissue transfers, consisting of either tendon or periosteal flaps, may be used to reinforce the superior peroneal retinaculum. However, these procedures sacrifice normal structures and potentially weakening them, and complications, such as sural nerve injury and ankle instability, have been reported [1, 5, 14].

If an anatomical approach is preferred to treat this pathology, reattachment of the superior peroneal retinaculum seems the most appropriate technique [5]. Both isolated retinaculum repair and its combination with groove-deepening procedures have been successful treatment options, demonstrating good to excellent outcomes, high satisfaction rates, and encouraging times and rates of return to sports [3, 12].

This case demonstrates the success of employing isolated reattachment of the superior peroneal retinaculum in peroneal tendon subluxation. Additionally, it encourages consideration of this pathology as a diagnostic possibility in both traumatic and atraumatic cases of ankle injuries, intending to avoid misdiagnosis or underdiagnosis, treatment failure, and injury reoccurrence.

## Data Availability

Not applicable.
